# A 4‐mm‐Short Implant Rehabilitation in the Posterior Maxilla With Dynamic Navigation Technology: A Case Report After 10 Years of Postloading Follow‐Up

**DOI:** 10.1155/crid/9591954

**Published:** 2026-08-07

**Authors:** Gerardo Pellegrino, Fabio Rossi, Andrea Oliverio, Subhi Tayeb, Claudia Angelino, Carlo Barausse, Pietro Felice

**Affiliations:** ^1^ Unit of Oral Surgery, Department of Biomedical and Neuromotor Sciences (DIBINEM), University of Bologna, Bologna, Italy, unibo.it; ^2^ Department of Health Sciences, Magna Graecia University of Catanzaro, Catanzaro, Italy, unicz.it; ^3^ Private Practice, Bologna, Italy

**Keywords:** case report, dynamic navigation, long-term follow-up, posterior maxilla, short dental implants

## Abstract

Implant rehabilitation in the posterior maxilla with severe vertical bone atrophy remains clinically challenging, particularly when bone augmentation procedures are not feasible or are refused by the patient. The use of ultrashort implants combined with dynamic navigation has been proposed as a minimally invasive alternative in selected cases; however, long‐term clinical evidence in the maxilla is still limited. This case report describes the 10‐year clinical and radiographic outcomes of a single 4‐mm ultrashort implant placed in the posterior maxilla using dynamic navigation technology. A 47‐year‐old nonsmoking female patient presented with a single edentulous maxillary first molar and severe vertical bone resorption (≤ 5 mm). The patient declined bone augmentation and fixed partial denture therapy. Implant placement was planned and performed under dynamic navigation guidance, followed by restoration with a single implant‐supported crown. Clinical and radiographic follow‐up was conducted regularly up to 10 years after prosthetic loading. Throughout the follow‐up period, no biological or prosthetic complications were observed. Peri‐implant soft tissues remained healthy, with no bleeding on probing or suppuration. Radiographic evaluation using standardized periapical radiographs revealed a mean marginal bone loss of 0.35 mm at 10 years. The implant‐supported crown maintained stable function and structural integrity, and the patient reported high satisfaction with the treatment outcome. Within the limitations of a single case report, these findings suggest that the combination of dynamic navigation and ultrashort implants may represent a reliable and minimally invasive treatment option for single implant rehabilitation in severely atrophic posterior maxillary sites. Further prospective studies with larger sample sizes and long‐term follow‐up are required to confirm the predictability and reproducibility of this approach.

## 1. Introduction

Implant rehabilitation in the posterior regions of atrophic jaws represents one of the most challenging clinical scenarios in oral surgery. Early loss of teeth frequently leads to progressive bone atrophy [1], resulting in reduced vertical bone height and difficulty in placing implants safely in relation to critical anatomical structures. Correct implant positioning is a fundamental prerequisite for long‐term success, ensuring anatomical preservation, an adequate prosthetic emergence profile, stable peri‐implant soft tissues, and reduced biological and mechanical complications [2, 3]. In this context, computer‐assisted implant surgery has been introduced to improve the transfer of the virtual plan to the surgical field, mainly through static guided surgery and dynamic navigation (DN) [4–6]. Static templates and DN present different operative characteristics, advantages, and limitations, particularly in terms of surgical visibility, irrigation, tactile feedback, and intraoperative flexibility [7, 8]. DN allows real‐time visualization of the drill and implant trajectory on CBCT‐based three‐dimensional imaging and may support implant placement in anatomically demanding sites [9–11]. Compared with static guidance, DN preserves intraoperative tactile feedback and allows the clinician to adjust the osteotomy when necessary [12, 13]. Its accuracy for implant placement has been reported in several clinical and preclinical studies, with deviations generally around 1 mm between planned and actual implant positions [11, 14, 15]. However, in the context of a single case report, DN should be considered a surgical aid for accurate placement rather than an independent determinant of long‐term implant success. Parallel to these technological developments, the use of short and ultrashort implants, defined as implants ≤ 6 mm in length [16–19], has progressively gained acceptance as a less invasive alternative to bone augmentation procedures, reducing morbidity, costs, and treatment time compared with conventional implants placed in regenerated bone [20, 21]. Recent studies and systematic reviews have reported high survival rates for short implants over medium‐ to long‐term follow‐up periods [22–25], with survival percentages ranging from 90% to 98% for implants ≤ 6 mm in posterior sites, yielding outcomes comparable to standard‐length implants. The introduction of 4‐mm ultrashort implants has further expanded rehabilitation options in severely atrophic jaws [18, 26]. Although initially employed mainly in the mandible, recent evidence suggests that, under appropriately selected conditions and with meticulous planning, these implants may also serve as a valid alternative to regenerative procedures in the maxilla [27]. No significant differences in survival rates have been reported between extrashort (4 mm) and long implants, although the available data cited primarily concern implants placed in the mandible; moreover, after 12 months of function, extrashort implants showed significantly lower marginal bone loss (MBL) (*p* = 0.003) compared with implants longer than 8 mm [28]. However, the limited residual bone height in these cases makes extremely precise positioning and adequate primary stability essential, as even minimal angular deviations may increase mechanical stresses on the implant and supporting bone, particularly in the presence of high crown‐to‐implant (C/I) ratios [29, 30]. Several reviews have reported that DN may improve three‐dimensional placement accuracy by reducing coronal, apical, and angular deviations, as well as the risk of damaging adjacent anatomical structures [31–33]. In severely atrophic sites treated with ultrashort implants, this technology may therefore help the clinician respect anatomical boundaries and control osteotomy preparation, whereas the long‐term outcome remains influenced by multiple biological, prosthetic, occlusal, and patient‐related factors [11, 34]. Therefore, the present case report focuses primarily on the long‐term clinical and radiographic performance of a 4‐mm ultrashort implant placed in a severely atrophic posterior maxillary site, where long‐term evidence remains limited. The present report describes the clinical and radiographic outcomes at 10 years of follow‐up of the rehabilitation of a patient with atrophic alveolar ridge in the region of maxillary first molar using a 4‐mm ultrashort implant placed with DN, extending the results of a previous 3‐year evaluation [35].

## 2. Case Presentation

The patient was treated at the Unit of Oral Surgery, Department of Biomedical and Neuromotor Sciences (DIBINEM) at the University of Bologna, Italy. The objectives and procedures of the study were thoroughly explained to the patient, and informed consent was obtained. The study was conducted in adherence to the Declaration of Helsinki and was prepared in accordance with the CARE (CAse REport) guidelines to ensure completeness and transparency in clinical reporting. The completed CARE checklist is provided as Supporting Information 1. The Local Ethics Committee of the University of Bologna was informed of the study; according to institutional policies, formal ethical approval was not required for this case report involving anonymized data.

A 47‐year‐old female patient, nonsmoker with no relevant systemic conditions, presented with the need to rehabilitate a single edentulous site corresponding to the maxillary right first molar. Clinical and radiographic examinations, including periapical radiographs and cone beam computed tomography (CBCT), revealed severe vertical bone resorption with a residual bone height of ≤ 5 mm (Figure 1). The patient expressed a clear preference for a minimally invasive approach to be completed in a single surgical session and refused any type of bone augmentation procedure. She also declined a fixed partial denture, not wishing to involve the adjacent teeth.

**Figure 1:**
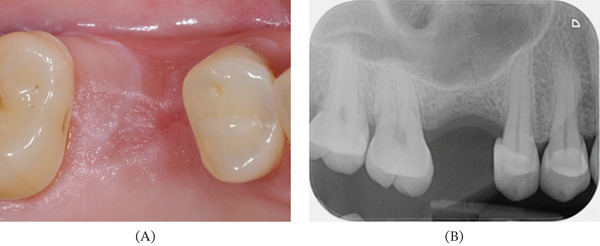
Preoperative clinical and radiographic assessment. (A) Preoperative intraoral view showing the edentulous site corresponding to the maxillary right first molar, with reduced alveolar ridge height. (B) Preoperative periapical radiograph confirming severe vertical bone resorption at the edentulous site, with a residual bone height ≤ 5 mm beneath the maxillary sinus floor. Reproduced from Pellegrino et al. [35], licensed under the Creative Commons Attribution (CC BY 4.0) license.

Given these conditions, rehabilitation with a 4‐mm‐long and 4.1‐mm‐wide ultrashort implant was proposed. The advantages of using DN to optimize implant placement accuracy and preserve the residual bone were also explained. The patient provided written informed consent after receiving a detailed description of the risks, benefits, and alternative treatment options.

### 2.1. Planning and Navigation Protocol

The ImplaNav DN system (BresMedical, Sydney, Australia) was used for the procedure. First, a preoperative CBCT scan was acquired with a marker plate (MP) anchored to the patient′s remaining teeth using a customized tray filled with high‐density impression material (Ramitec, 3M ESPE, St. Paul, Minnesota, United States). The anchorage impression engaged three healthy maxillary teeth. After CBCT acquisition, the reference assembly was removed and stored to be repositioned during surgery.

The MP contained the fiducial markers required for the intraoperative registration process. DICOM data representing the hard tissue structures and STL (Standard Triangulation Language) files of the dental and soft tissue surfaces, obtained from scans of the wax‐up models, were merged using dedicated ImplaNav software to select the appropriate implant type and position according to anatomical and prosthetic requirements. The virtually planned implant was visible in real time during surgery.

During the surgical procedure, a registration/calibration process was performed to allow the software to match the preoperative CBCT, containing the virtual implant position, with the surgical instruments used intraorally. The MP with fiducial markers was repositioned exactly as during the CBCT acquisition, enabling the system to align the markers in the CBCT dataset with those on the patient. A patient reference frame (pRF) was attached to the MP, whereas a second reference frame (hRF) was secured to the surgical handpiece. Both reference frames carried three reflective spheres arranged in different geometries. An infrared camera (NDI Polaris Vicra, Northern Digital, Waterloo, Ontario, Canada) detected the spatial position of both reference frames simultaneously, allowing the system to track the position of the patient and the handpiece and display the drill trajectory on the patient′s CBCT images.

To complete the registration/calibration process, the operator exposed the hRF attached to the handpiece to the camera for at least 3 s, allowing it to be matched with the pRF. The fiducial markers on the MP were then touched sequentially with a calibration drill of known length. Once this step was completed, no further calibration was required for the subsequent drills already stored in the digital library. The software was thus able to superimpose the position and angulation of the surgical instrument onto the patient′s preoperative 3D radiological images [34, 36, 37] (Figure 2).

**Figure 2:**
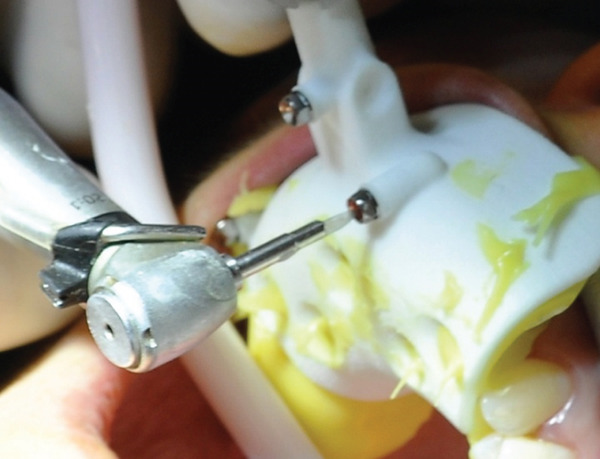
The registration/calibration just before the surgical procedure.

### 2.2. Surgical and Prosthetic Procedure

After local anesthesia with two cartridges of 2% mepivacaine with epinephrine 1:50,000, corresponding to a total mepivacaine dose of 72 mg—administered as one cartridge by buccal infiltration and one cartridge by palatal infiltration—a minimal crestal incision without vertical releasing incisions was performed. The osteotomy was prepared under DN guidance, and a 4‐mm‐long, 4.1‐mm‐wide SLActive tissue‐level implant (Institut Straumann AG, Basel, Switzerland) was placed according to the manufacturer′s guidelines (Figures 3, 4, and 5). The implant achieved good primary stability with an insertion torque ≥ 35 Ncm. The wound was sutured with 4‐0 silk sutures.

**Figure 3:**
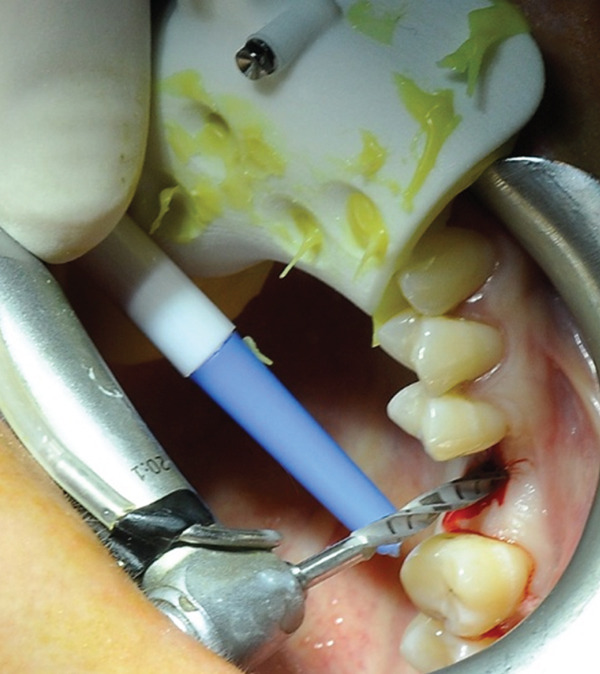
Clinical view of the implant site preparation.

**Figure 4:**
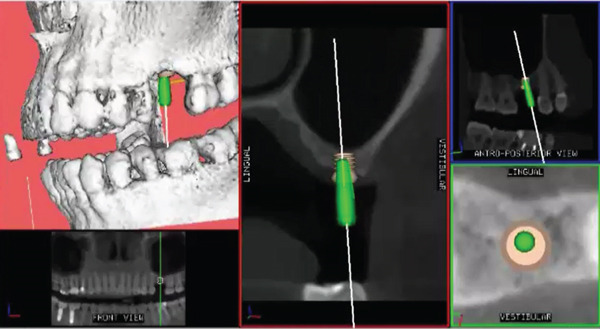
Dynamic navigation screen view displaying the virtually planned implant position, with the green marker representing the active drill superimposed on the patient′s three‐dimensional radiological anatomy in real time.

**Figure 5:**
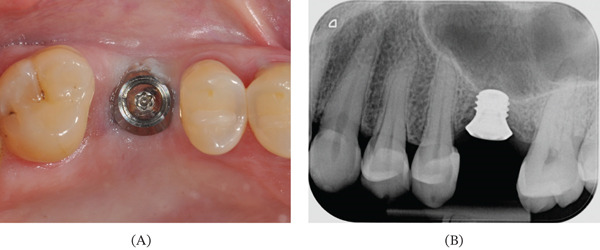
Postoperative clinical and radiographic outcomes. (A) Intraoral postoperative view showing the ultrashort implant in situ with healthy peri‐implant soft tissues and adequate transmucosal emergence. (B) Standardized periapical radiograph acquired after implant placement, showing accurate implant positioning and the close relationship with the maxillary sinus floor. Reproduced from Pellegrino et al. [35], licensed under the Creative Commons Attribution (CC BY 4.0) license.

Postoperative instructions included adherence to a soft diet, standard oral hygiene measures, and rinsing with a 0.2% chlorhexidine mouthwash. No postoperative discomfort—such as bleeding, edema, or pain—was reported, and no complications occurred.

After 6 weeks, a straight conical abutment was connected, and impressions were taken using a customized tray. A provisional acrylic resin crown was delivered 1 week later and replaced with the definitive ceramic crown after 3 months. Considering that the restoration was supported by a single 4‐mm ultrashort implant in the posterior maxilla, with an increased C/I ratio, the definitive prosthesis was intentionally designed in slight infraocclusion as a protective prosthetic strategy. The final prosthesis was designed to minimize lateral and protrusive contacts while maintaining only light and stable axial contacts in maximum intercuspation, in order to reduce nonaxial forces on the ultrashort implant. The patient attended professional hygiene sessions every 3 months for the entire follow‐up period.

### 2.3. Follow‐up and Outcomes

The patient attended all scheduled recall visits up to the 10‐year follow‐up. Throughout this period, no surgical or prosthetic complications were observed. Clinically, the peri‐implant soft tissues remained healthy, with no bleeding on probing, no suppuration, and a stable mucosal collar around the implant (Figures 6, 7, 8, and 9).

**Figure 6:**
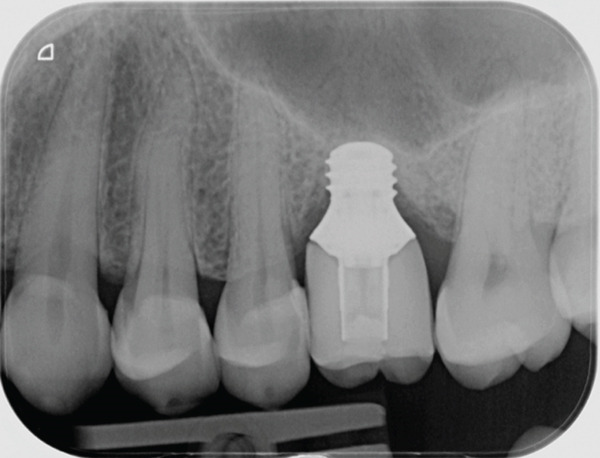
Periapical radiograph at 1‐year follow‐up after prosthetic loading, showing stable marginal bone levels around the ultrashort implant.

**Figure 7:**
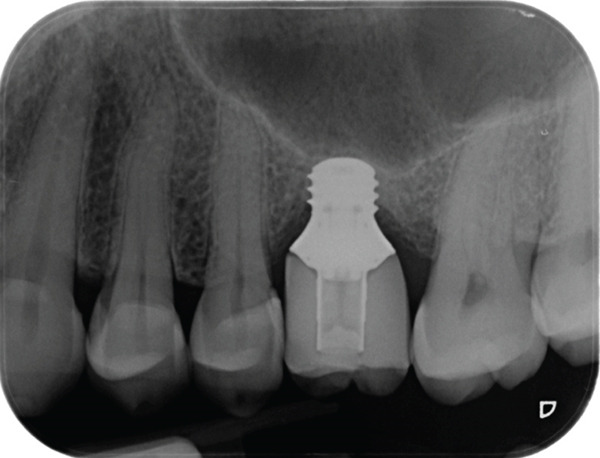
Periapical radiograph at 5‐year follow‐up after prosthetic loading, showing stable marginal bone levels around the ultrashort implant.

**Figure 8:**
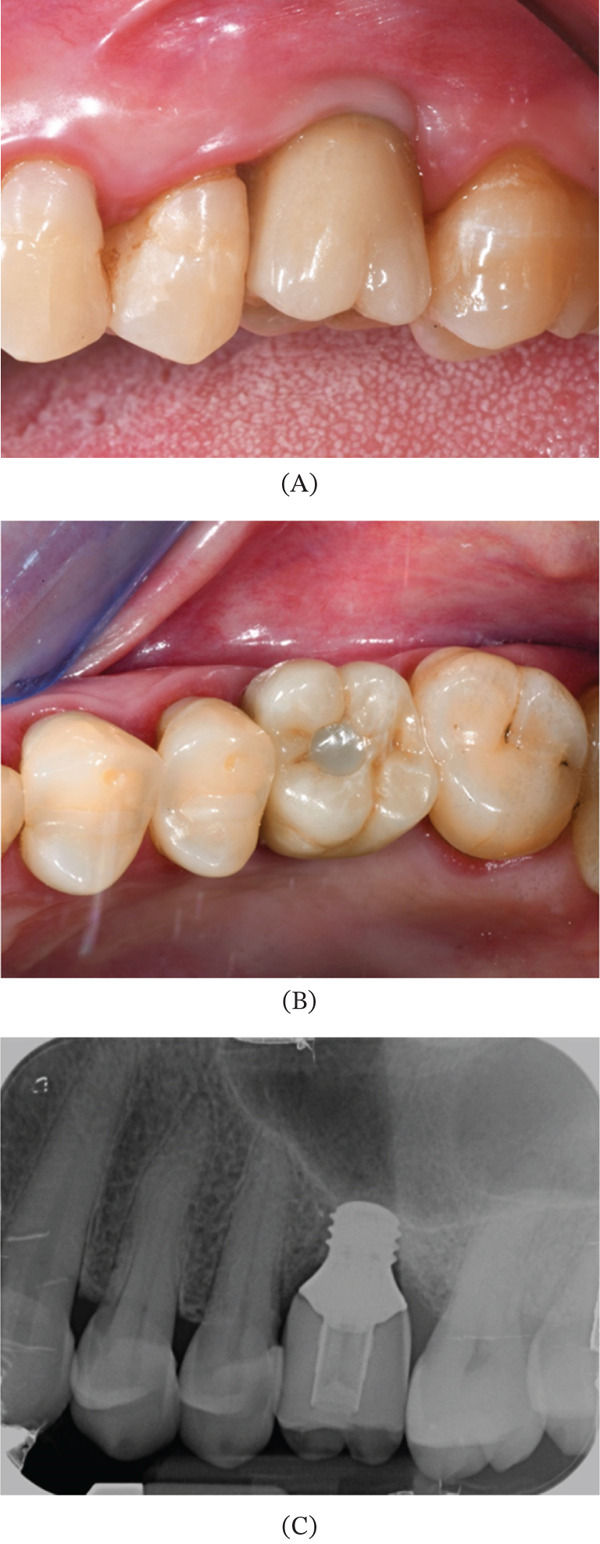
Clinical and radiographic evaluation at the 10‐year follow‐up. (A) Intraoral view showing healthy peri‐implant soft tissues and a stable mucosal profile around the implant‐supported crown. (B) Occlusal view confirming adequate integration of the restoration within the occlusal scheme, with no signs of functional overload. (C) Standardized periapical radiograph indicating stable peri‐implant bone levels, with no radiographic signs of progressive marginal bone loss compared with baseline at prosthetic loading.

**Figure 9:**
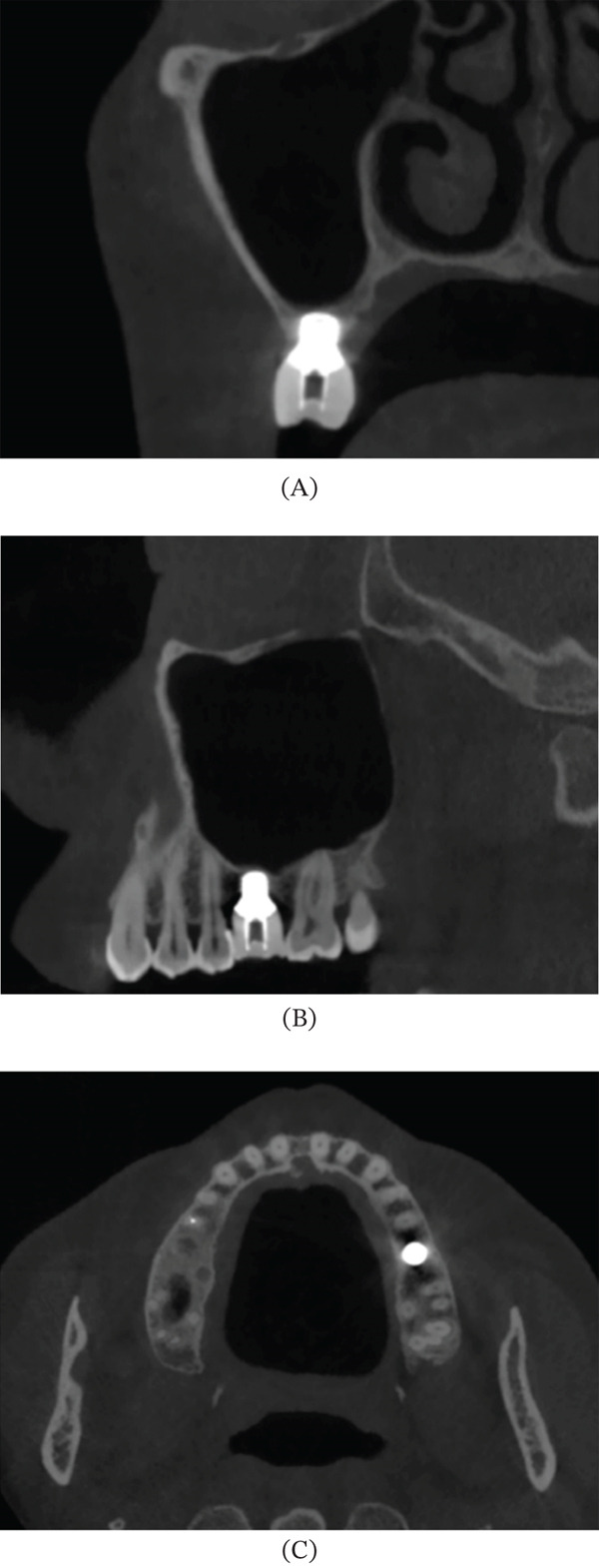
Cone beam computed tomography (CBCT) images obtained at the 10‐year follow‐up. (A) Parasagittal view showing stable peri‐implant bone conditions in relation to the maxillary sinus floor. (B) Cross‐sectional view illustrating the implant position within the residual alveolar ridge and the surrounding bone contours. (C) Axial view depicting the buccopalatal implant positioning and preservation of the peri‐implant bone envelope.

For marginal bone level assessment, each image was calibrated using the known length of the implant as a reference, allowing standardized and reproducible linear measurements. Periapical radiographs were obtained at each follow‐up visit and analyzed using dedicated software (Weasis DICOM medical viewer, Weasis Team).

Radiographic evaluations performed throughout the follow‐up period revealed a progressive but limited mean MBL, measuring 0.15 mm at 1 year and 0.25 mm at 3 years, remaining stable at 5 years, and reaching 0.35 mm at 10 years. The C/I ratio of the definitive restoration, calculated on calibrated periapical radiographs as the ratio between the prosthetic crown height and the implant length, was approximately 2.5:1.

The ceramic crown maintained excellent function and structural integrity throughout the follow‐up period, with no evidence of chipping, screw loosening, or need for repair.

The treatment objectives were successfully achieved with stable occlusal contacts, and the patient reported complete satisfaction in terms of comfort, function, and overall treatment outcome.

## 3. Discussion

Rehabilitation of the posterior regions of atrophic jaws using short and ultrashort implants currently represents a well‐established strategy supported by an increasing body of clinical evidence. The ITI Consensus Report Group 1 (2018) and the EAO Consensus Conference Group 4 (2015), together with numerous clinical studies and systematic reviews, have reported that implants with a length ≤ 6 mm can achieve high survival rates in the medium to long term, generally ranging between 90% and 98%, with clinical outcomes comparable to those of conventional implants placed with more invasive regenerative procedures, such as maxillary sinus floor elevation or vertical mandibular augmentation [19, 22, 26, 37–41].

Recent multicenter randomized controlled trials and meta‐analyses have further confirmed the equivalence, in terms of implant survival and MBL, between short implants (6 mm) and standard‐length implants (11–15 mm) placed in regenerated sites following lateral sinus floor elevation, with follow‐up periods extending from 5 to 10 years [24, 42, 43].

In recent years, attention has increasingly shifted toward 4‐mm ultrashort implants, which were initially used predominantly in the posterior mandible. Prospective and retrospective studies have reported survival rates ranging approximately from 92% to 98% during the early follow‐up period, both in pristine sites and in sites previously affected by regenerative procedure failures [18, 28, 44]. More recently, a large retrospective study investigating 4‐mm extrashort implants in atrophic posterior jaws documented a cumulative survival rate of 95.36% at a mean follow‐up slightly exceeding 8 years, with limited MBL and a low incidence of biological and prosthetic complications [26]. Although these data remain more limited than those available for standard‐length implants, they suggest that 4‐mm implants may represent a valid alternative to bone augmentation procedures in carefully selected cases.

Nevertheless, recent systematic reviews have highlighted that, at the meta‐analytic level, short implants may exhibit a slightly higher risk of failure compared with longer implants while still maintaining overall favorable and clinically acceptable outcomes [45, 46]. In particular, bone quality, occlusal load control, and appropriate implant site preparation have been emphasized as critical variables influencing long‐term success, especially when working with severely reduced residual bone height.

One aspect traditionally considered problematic in short implants is the C/I ratio, which is often increased in the presence of severe vertical atrophy. However, several medium‐ to long‐term clinical follow‐up studies have not identified a clear association between unfavorable C/I ratios and increased MBL, provided that occlusal loads are predominantly axial and adequate plaque control is maintained [47–49]. It has also been observed that 4‐mm extrashort implants may exhibit—at least in the short term, significantly lower MBL compared with longer implants—together with fewer biological and prosthetic complications, despite the absence of significant differences in survival rates after 12 months of function [28]. These findings reinforce the concept that the critical factor is not so much the implant length itself, but rather the orientation of functional loading, together with accurate implant positioning and maintenance of peri‐implant health.

DN was used in the present case as a surgical aid to support accurate three‐dimensional implant placement in a site with markedly reduced residual bone height. Previous systematic reviews and clinical studies have reported that DN may reduce coronal, apical, and angular deviations compared with free‐hand placement, with accuracy levels comparable to those of static guided surgery [3, 48, 50]. Additionally, Wu et al. [51] suggested that different registration protocols may provide comparable accuracy in posterior regions, and that navigation‐assisted procedures can be useful when surgical access or anatomical safety margins are limited [52, 53]. Nevertheless, the present case report cannot establish a causal relationship between the use of DN and the favorable 10‐year outcome. The long‐term stability observed in this patient should therefore be interpreted as the result of multiple factors, including careful case selection, implant surface characteristics, primary stability, prosthetic design, occlusal control, and supportive maintenance, rather than as evidence of an independent effect of DN on implant longevity.

In the present case, the placement of a 4‐mm ultrashort implant in the posterior maxilla represented the only feasible implant‐supported option in the absence of bone augmentation procedures, in order to meet the patient′s expectations. Achieving primary stability and maintaining bone height appeared challenging, highlighting the need for precise implant site preparation that respected the available bone height while accounting for prosthetic loading. The surface properties of the implant increased the bone‐to‐implant contact area and contributed to achieving immediate primary stability. Moreover, precise implant positioning addressed the need to avoid any implant inclination, thereby preventing lateral forces, particularly in consideration of the prosthetic crown length and a C/I ratio ≥ 2.5. The absence of surgical and prosthetic complications, the clinical stability of peri‐implant soft tissues (with no bleeding on probing or signs of mucositis), and the lack of clinically relevant MBL on radiographic evaluation support the hypothesis that, in properly selected cases and with strict three‐dimensional control of implant positioning, 4‐mm implants may represent a reliable solution even in the posterior maxilla.

The slightly infraoccluded position of the implant‐supported restoration visible in the clinical photographs should also be considered when interpreting the long‐term outcome. Given the limited availability of long‐term follow‐up data on single ultrashort implants placed in atrophic posterior maxillary sites, this design was adopted as a precautionary prosthetic strategy. The definitive crown was planned to minimize lateral and protrusive contacts while maintaining stable axial contacts in maximum intercuspation, as described in the prosthetic protocol. This occlusal scheme was maintained and periodically checked during the follow‐up visits. For this reason, the favorable 10‐year outcome should be interpreted in the context of a carefully controlled occlusal scheme rather than generalized to all ultrashort implants subjected to different loading conditions.

In addition to implant‐related, surgical, and prosthetic factors, patient compliance may have contributed to the favorable long‐term outcome observed in this case. The patient attended all scheduled recall visits and professional maintenance appointments every 6 months throughout the follow‐up period, allowing regular reinforcement of oral hygiene measures, peri‐implant tissue monitoring, and occlusal control. This strict supportive care may have helped maintain peri‐implant health and reduce the risk of biological and mechanical complications over time.

The extension of the follow‐up to 10 years is of particular relevance, as long‐term data (≥ 5 years) on short and extrashort implants in the maxilla remain limited and are predominantly derived from mandibular cohorts or prospective studies with heterogeneous populations [54–58]. Nevertheless, given the descriptive nature of a case report and the absence of a control group, the results presented in this study should be considered exploratory and hypothesis‐generating rather than conclusive.

Overall, the present case documents the 10‐year clinical and radiographic stability of a single 4‐mm ultrashort implant placed in a severely atrophic posterior maxillary site with the aid of DN. Within the limitations of a single case report, these findings suggest that ultrashort implants may represent a minimally invasive option in carefully selected patients who refuse or are not candidates for bone augmentation procedures. However, further well‐designed prospective controlled studies with larger sample sizes and extended follow‐up are required to clarify the long‐term predictability of this approach and the relative contribution of surgical guidance, prosthetic design, occlusal loading, and patient‐related factors to treatment outcomes.

## 4. Conclusions

This case report describes the 10‐year clinical and radiographic outcomes of a single 4‐mm ultrashort implant placed in the posterior maxilla using DN technology. Over the entire follow‐up period, the implant‐supported restoration remained clinically stable, with healthy peri‐implant soft tissues, absence of biological or prosthetic complications, and limited MBL.

Within the limitations of a single‐patient case report, the present findings suggest that the use of DN may facilitate accurate implant placement in severely atrophic posterior maxillary sites, allowing the application of ultrashort implants as a minimally invasive treatment option when bone augmentation procedures are not feasible or are refused by the patient.

Further clinical investigations with larger sample sizes and long‐term follow‐up are necessary to confirm the reproducibility and long‐term outcomes of this approach.

## Author Contributions

Conceptualization: G.P. and P.F. Methodology: G.P. and F.R. Validation: G.P. and F.R. Investigation: A.O. and C.A. Data curation: S.T. and A.O. Writing—original draft preparation: A.O. and S.T. Writing—review and editing: C.B. and G.P. Supervision: G.P. and P.F. Project administration: P.F.

## Funding

No funding was received for this manuscript.

## Consent

The patient has been adequately informed about the treatment, understands the risks and benefits, and consents to proceed. The patient also consents to the use of photographs and radiographic exams for scientific and educational purposes, authorizing their anonymous publication. The informed consent has been signed.

## Conflicts of Interest

The authors declare no conflicts of interest.

## Supporting information


**Supporting Information** Additional supporting information can be found online in the Supporting Information section. Supporting Information. File S1:CARE‐checklist provided along with the manuscript.

## Data Availability

The data used to support the findings of this study are available from the corresponding author upon request.
