# Supplementary material for: A 4‐mm‐Short Implant Rehabilitation in the Posterior Maxilla With Dynamic Navigation Technology: A Case Report After 10 Years of Postloading Follow‐Up
Source: Case Rep Dent. 2026 Aug 7;2026:9591954. doi: 10.1155/crid/9591954 (PMC13451833; doi:10.1155/crid/9591954)
Supplement: Supplementary file 1 [file CRID-2026-9591954-s001.docx]

**CARE Checklist of information to include when writing a case report**

**Topic	Item	Checklist item description	Reported on Line**

**Title	1**	The diagnosis or intervention of primary focus followed by the words “case report”	1

**Key Words	2**	2 to 5 key words that identify diagnoses or interventions in this case report, including "case report"	2

# Abstract

**(no references)**

**3a**	Introduction: What is unique about this case and what does it add to the scientific literature?	2

**3b**	Main symptoms and/or important clinical findings	2

**3c**	The main diagnoses, therapeutic interventions, and outcomes	2

**3d**	Conclusion—What is the main “take-away” lesson(s) from this case?	2

**Introduction	4**	One or two paragraphs summarizing why this case is unique (**may include references**)	3

**Patient Information	5a**	De-identified patient specific information	4

**5b**	Primary concerns and symptoms of the patient	4

**5c**	Medical, family, and psycho-social history including relevant genetic information	4

**5d**	Relevant past interventions with outcomes	N/A

# Clinical Findings

**Timeline**

**Diagnostic Assessment**

**Therapeutic Intervention**

**Follow-up and Outcomes**

6 Describe significant physical examination (PE) and important clinical findings	4
7 Historical and current information from this episode of care organized as a timeline	N/A

**8a**	Diagnostic testing (such as PE, laboratory testing, imaging, surveys).	4

**8b**	Diagnostic challenges (such as access to testing, financial, or cultural)	N/A

**8c**	Diagnosis (including other diagnoses considered)	4

**8d**	Prognosis (such as staging in oncology) where applicable	N/A

**9a**	Types of therapeutic intervention (such as pharmacologic, surgical, preventive, self-care)	5

**9b**	Administration of therapeutic intervention (such as dosage, strength, duration)	5

**9c**	Changes in therapeutic intervention (with rationale)	N/A

**10a**	Clinician and patient-assessed outcomes (if available)	6

**10b**	Important follow-up diagnostic and other test results	6

**10c**	Intervention adherence and tolerability (How was this assessed?)	5-6

**10d**	Adverse and unanticipated events	6

**Discussion	11a**	A scientific discussion of the strengths AND limitations associated with this case report	7-8

**11b**	Discussion of the relevant medical literature **with references**	6-8

**11c**	The scientific rationale for any conclusions (including assessment of possible causes)	7-8

**11d**	The primary “take-away” lessons of this case report (without references) in a one paragraph conclusion	8

**Patient Perspective	12**	The patient should share their perspective in one to two paragraphs on the treatment(s) they received	N/A

**Informed Consent	13**	Did the patient give informed consent? Please provide if requested . . . . . . . . . . . . . . . . . . . . . . . . . . . . . . . . . . . . . .	**Yes	X	No**
